# Simultaneous Determination of Polyamines and Steroids in Human Serum from Breast Cancer Patients Using Liquid Chromatography–Tandem Mass Spectrometry

**DOI:** 10.3390/molecules26041153

**Published:** 2021-02-21

**Authors:** Yu Ra Lee, Ji Won Lee, Jongki Hong, Bong Chul Chung

**Affiliations:** 1 Molecular Recognition Research Center, Korea Institute of Science and Technology, Seoul 02792, Korea; T16627@kist.re.kr; 2 KHU-KIST Department of Converging Science and Technology, Kyung Hee University, Seoul 02447, Korea; 3 Department of Family Medicine, Gangnam Severance Hospital, Yonsei University College of Medicine, Seoul 06273, Korea; indi5645@yuhs.ac; 4 College of Pharmacy, Kyung Hee University, Seoul 02447, Korea

**Keywords:** polyamine, steroid, breast cancer, liquid chromatography–tandem mass spectrometry, serum

## Abstract

A simultaneous quantitative profiling method for polyamines and steroids using liquid chromatography–tandem mass spectrometry was developed and validated. We applied this method to human serum samples to simultaneously evaluate polyamine and steroid levels. Chemical derivatization was performed using isobutyl chloroformate to increase the sensitivity of polyamines. The method was validated, and the matrix effects were in the range of 78.7–126.3% and recoveries were in the range of 87.8–123.6%. Moreover, the intra-day accuracy and precision were in the ranges of 86.5–116.2% and 0.6–21.8%, respectively, whereas the inter-day accuracy and precision were in the ranges of 82.0–119.3% and 0.3–20.2%, respectively. The linearity was greater than 0.99. The validated method was used to investigate the differences in polyamine and steroid levels between treated breast cancer patients and normal controls. In our results, *N*-acetyl putrescine, *N*-acetyl spermidine, cadaverine, 1,3-diaminopropane, and epitestosterone were significantly higher in the breast cancer patient group. Through receiver operating characteristic curve analysis, all metabolites that were significantly increased in patient groups with areas under the curve >0.8 were shown. This mass spectrometry-based quantitative profiling method, used for the investigation of breast cancer, is also applicable to androgen-dependent diseases and polyamine-related diseases.

## 1. Introduction

Breast cancer is a common hormone-related cancer, which includes estrogen receptor-positive and progesterone receptor-positive disease. According to statistics from 2017, it is the fifth most common type of cancer in Korea and ranks fifth in cancer-related mortality. In addition, breast cancer ranks first among women as a cause of death from cancer [1]. Although the incidence of most cancers in Korean women has been declining since 2007, breast cancer continues to increase, with the highest incidence among other cancer types. Despite an increasing understanding of the molecular etiology of breast cancer over the past 20 years, there remains a lack of reliable biomarkers to monitor treatment efficacy associated with the disease. Currently, treatment of breast cancer commonly involves surgery, chemotherapy, radiotherapy, and hormone therapy [2]. However, it is important to confirm the effect of breast cancer treatment with a simple experimental method.

Polyamines are aliphatic amines composed of straight chains of carbon atoms. Amines have several biological implications, particularly polyamines, which mainly act as proliferation factors in cells. Cancer cells are highly proliferative; therefore, polyamines are one of the most important biomarkers in cancer research. The increase in polyamine concentrations in urine samples from patients with malignant cancer was first reported by D. H. Russell in 1971 [3]. Since then, polyamines have been analyzed in various biological fluids to investigate their potential as markers for the early diagnosis of cancer, evaluation of progression, and prediction of disease recurrence. It can also be seen that the number of polyamines is distributed differently depending on the type of lung and liver cancer [4]. In breast cancer patients, it has been reported that acetylated polyamines are present at a much higher concentration compared to that in normal human breast tissue [5,6]. Moreover, altered polyamines can be useful markers for the evaluation of breast cancer treatment efficacy [7].

It has been suggested that hormones such as progesterone, estrogens, and androgens are implicated in the development and/or growth of normal and neoplastic mammary tissue. Androgens, which play an important role in the development of prostate cancer, have also been shown to be associated with breast cancer, attracting academic attention. This is because the growth of estrogen receptor-positive breast cancer is decreased after blocking the androgen receptor [8]. In addition, androgens have been proposed to control tumor growth rates [9]. Free testosterone, an androgen, might play an important role in the development of breast cancer in women [10]. One study found that young women with high levels of a male hormone, like androgens, have a higher risk of developing breast cancer [11]. Progesterone, a female hormone, is also associated with breast cancer. In particular, progesterone receptors in breast cancer cells interact with estrogen receptors to change their mode of action and delay tumor growth [12]. This study suggests that an overall evaluation of polyamines and steroids will provide information on breast cancer treatment. Therefore, profiling the combined metabolism of polyamines and steroids is required in breast cancer patients.

Metabolic approaches can monitor an individual’s status and help detect potential cancer biomarkers [13]. Gas chromatography–mass spectrometry and liquid chromatography–mass spectrometry (LC-MS) are used for quantitative metabolic profiling, as mass spectrometry delivers high sensitivity and is capable of characterizing complex biological samples [14,15]. In particular, in the case of cancer patients, since normal cells are mutated, not only free polyamines but also acetylated polyamines must be analyzed in relation to intracellular metabolic pathways. However, while analyzing acetylated polyamines using gas chromatography–mass spectrometry, additional active hydrogen must be removed, which requires additional derivatization [16]. One disadvantage of this is that the experiment time is increased due to the use of two or more derivatizations to remove active hydrogen sites. This process makes it less suitable for the analysis of acetylated polyamines. In addition, more specific and sensitive results can be obtained when using liquid chromatography–tandem mass spectrometry (LC-MS/MS) than LC-MS, which can be carried out using one derivatization, and thus, we conducted an experiment using the LC-MS/MS condition.

Therefore, in this study, an analytical method was validated for the simultaneous quantitative profiling of serum polyamines and steroids. To the best of our knowledge, this study is the first to simultaneously analyze polyamines and steroids in human serum samples from breast cancer patients. The developed and validated method was applied to analyze the serum concentrations of nine polyamines and eight steroids in patients with breast cancer after treatment and in normal female subjects using LC-MS/MS system.

## 2. Results and Discussion

### 2.1. Sample Preparation and Optimization

Polyamines have a positive charge at physiological pH [17] due to amino groups in their molecular structure; therefore, they easily bind with other substances. However, they are low-molecular weight molecules that rapidly elute from the chromatogram and therefore hinder accurate analysis. To solve this problem, we used an amine-carbamylated derivatization agent, isobutyl chloroformate. Reaction with a derivatization reagent prioritizes the binding of polyamines to other substances, and, therefore, it is possible to selectively extract polyamines.

Nine polyamines that were detected in the biological sample were subjected to an amine-carbamylated derivatization reaction. After polyamine derivatization, deprotonation was difficult because the amine and carbamoyl groups formed a stable bond. In addition, acetyl polyamine was substituted with nitrogen to which no acetyl group was attached. As a result, one carbamoyl group was substituted in *N*-acetyl putrescine (N-PUT) and *N*-acetyl cadaverine (N-CAD), two carbamoyl groups in *N*-acetyl spermidine (N-SPD), and three carbamoyl groups in *N*-acetyl spermine (N-SPM).

**Figure 1:**
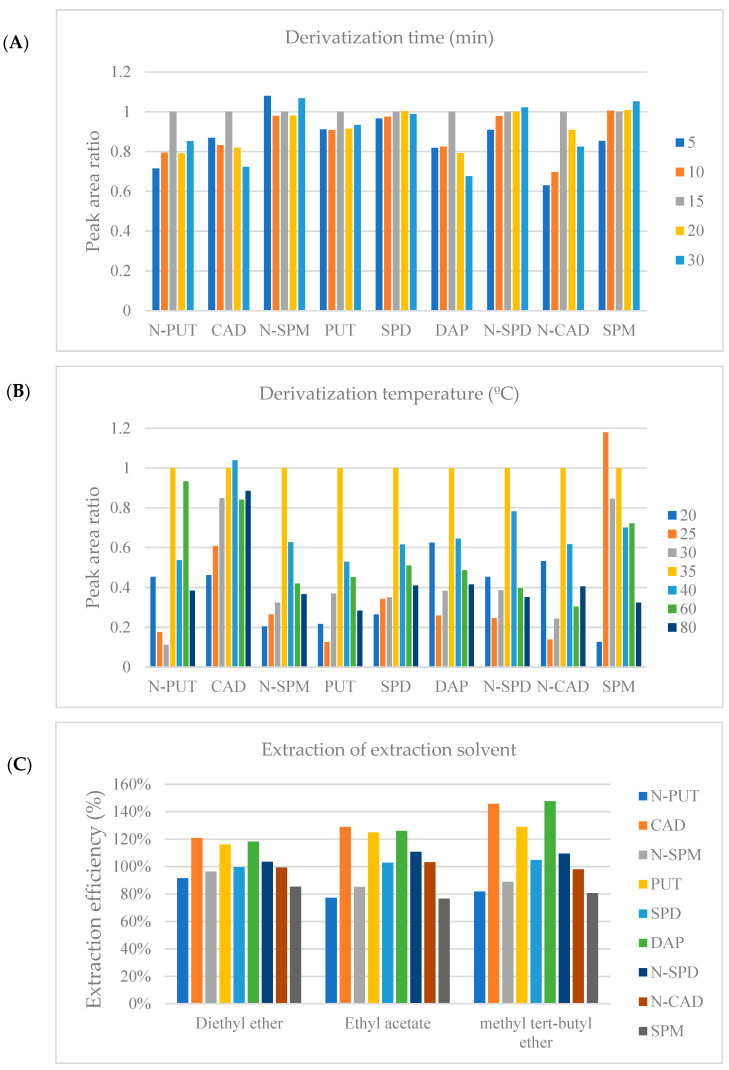
Comparison of the optimization procedures. (**A**) Isobutyl chloroformate derivatization under varying conditions including reaction time, (**B**) isobutyl chloroformate derivatization under varying reaction temperatures, and (**C**) extraction of liquid–liquid extraction solvents (N-PUT: *N*-acetyl putrescine; CAD: cadaverine; N-SPM: *N*-acetyl spermine; PUT: putrescine; SPD: spermidine; DAP: 1,3-diaminopropane; N-SPD: *N*-acetyl spermidine; N-CAD: *N*-acetyl cadaverine; SPM: spermine). The peak area ratios were expressed by dividing (**A**) 15 min and (**B**) 35 °C as standard values that we used for the conditions of this experiment.

The carbamylation procedure that has been routinely used in our laboratory for polyamine analysis [18] had to be optimized. Serum samples were precipitated by reactions at high temperature (60 °C) for 20 min, derivatized using isobutyl chloroformate, and extracted using the liquid–liquid extraction (LLE) method with diethyl ether. Derivatization conditions were optimized to improve analyte sensitivity. Optimum conditions were selected by comparing the peak areas. A reaction time of 15 min (Figure 1A) and a reaction temperature of 35 °C were selected (Figure 1B). The LLE method was used to clean the sample and minimize interference. Three different eluents, diethyl ether, ethyl acetate, and methyl tert-butyl ether (MTBE), were tested to enhance the extraction efficacy. The extraction efficiencies of polyamines were relatively higher in MTBE; however, those of cadaverine (CAD), PUT, and 1,3-diaminopropane (DAP) were outside the acceptable range. The extraction efficiencies of all analytes were acceptable when diethyl ether was used (Figure 1C). This optimization condition was the same as that in our previous experimental conditions [18].

### 2.2. Liquid Chromatography–Tandem Mass Spectrometry

**Figure 2:**
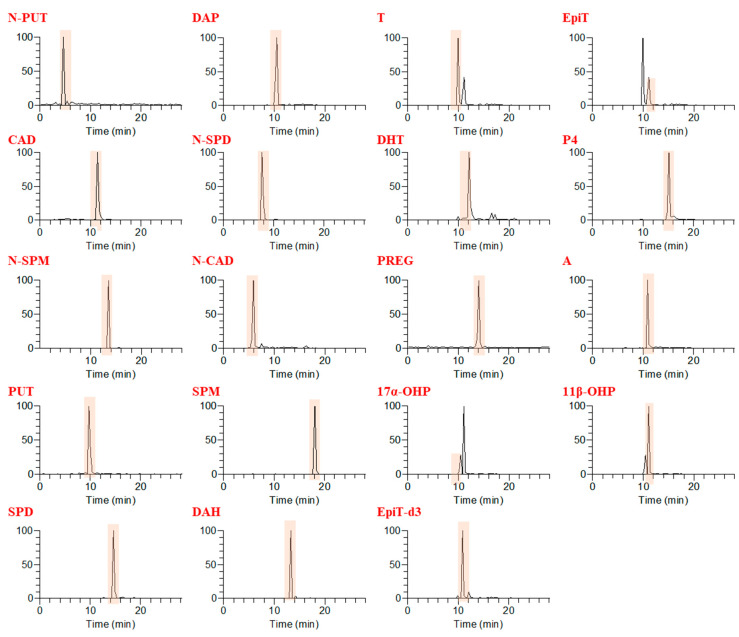
Chromatograms of nine polyamines, eight steroids, and two internal standards (IS) in the selected reaction monitoring mode, with target standard pretreatment (500 ng/mL). N-PUT: *N*-acetyl putrescine; CAD: cadaverine; N-SPM: *N*-acetyl spermine; PUT: putrescine; SPD: spermidine; DAP: 1,3-diaminopropane; N-SPD: *N*-acetyl spermidine; N-CAD: *N*-acetyl cadaverine; SPM: spermine; T: testosterone; EpiT: epitestosterone; DHT: dihydrotestosterone; PREG: pregnenolone; 17α-OHP:17α-hydroxyprogesterone; 11β-OHP: 11β-hydroxyprogesterone; A: androstenedione; P4: progesterone.

After the derivatization reaction, the analyte in the chromatogram can be effectively separated and analyzed simultaneously (Figure 2). In addition, when isobutyl chloroformate is used, the reaction in the aqueous solution is easy and exhibits high efficiency. This reaction can occur even at room temperature (20 to 25 °C) or with a little warming, with the completion of the derivatization reaction occurring in a short time (5 to 15 min).

**Figure 3:**
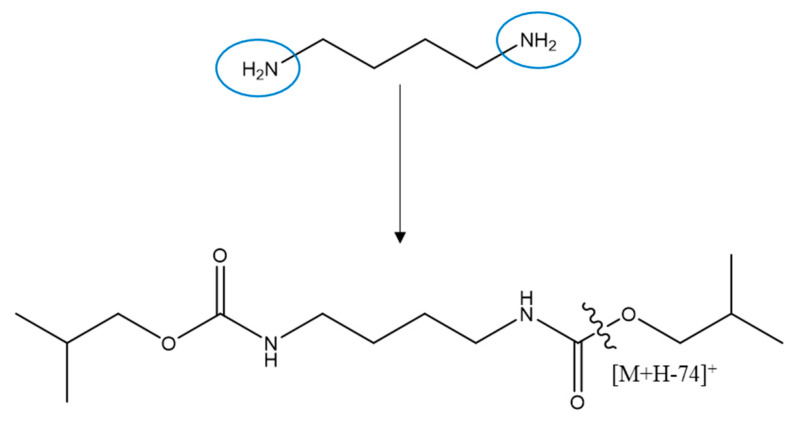
Fragmentation pattern of putrescine upon isobutyl chloroformate derivatization.

All polyamines and steroids produced protonated precursor ions [M + H]^+^ in the positive ion mode. All polyamines were derivatized with isobutyl chloroformate, whereas the steroids were not derivatized. Therefore, all steroids were detected in the free form, and steroid analysis revealed good intensity even without derivatization. All polyamines showed the [M + H − OCH_2_C_3_H_7_]^+^ ion as the base peak for quantitation (Figure 3). All steroids showed the [M + H − H_2_O]^+^ ion as the base peak of a fragment of high intensity for quantitation. In principle, LC-MS/MS using a stable internal standard (IS) is an optimal method for quantitative analysis. In particular, among our analytes, epimer-type substances (epitestosterone, testosterone, 17α-hydroxyprogesterone, and 11β-hydroxyprogesterone) might show false positives. However, we tried to separate the substances in the form of epimers as much as possible by adjusting the retention time, and there was a difference of approximately 0.6–1 min. Using the present method with aqueous extracts of serum, we achieved excellent separation of nine polyamines and eight steroids with no significantly interfering background peaks.

### 2.3. Method Validation

The developed method was validated by assessing the accuracy and precision of the quality control (QC) samples with four different concentrations. Moreover, linearity was performed with 10 calibration points (0.1, 1, 5, 10, 50, 100, 500, 1000, 2000, and 5000 ng/mL), excluding 5000 ng/mL and adding 0.5 ng/mL for 17α-hydroxyprogesterone (17α-OHP), 11β-hydroxyprogesterone (11β-OHP), androstenedione (A), and progesterone (P4). The regression equation was found to be linear over the dynamic ranges of all analytes (with correlation coefficient, R^2^ > 0.99). The limit of quantification (LOQ) values were determined for most polyamines at 1 ng/mL, except *N*-acetyl spermine (N-SPM; 0.1 ng/mL). In contrast, the LOQ values were determined for testosterone (T), epitestosterone (EpiT), dihydrotestosterone (DHT), and pregnenolone (PREG) at 1 ng/mL, and the LOQ values were determined for 17α-OHP, 11β-OHP, A, and P4 at 0.1 ng/mL. As shown in Table 1, we checked the matrix effect and overall recovery. Most analytes did not present any significant matrix effect, ranging from 78.7 to 126.3%, and the recovery of analytes was 87.8–123.6%. Precision was assessed based on the coefficient of variation (% CV), and accuracy was assessed through the relative error rate (% bias). For polyamine analysis, the intra-day (*n* = 3) precision (coefficient of variation (% CV)) and accuracy (% bias) were in the ranges of 1.6–21.2% and 86.5–116.2%, respectively, whereas the inter-day (*n* = 3) precision and accuracy were in the ranges of 0.3–20.2% and 87.8–119.3%, respectively. For steroid analysis, the intra-day (*n* = 3) precision (% CV) and accuracy (% bias) were in the ranges of 0.6–21.8% and 91–114.3%, respectively, whereas the inter-day (*n* = 3) precision and accuracy were in the ranges of 1.2–18.5% and 82–108.0%, respectively (Table 2).

### 2.4. Application of Serum Polyamine and Steroid Profiles to Patients with Breast Cancer and Normal Controls

In this study, we quantitatively analyzed polyamines and steroids in human serum from treated cancer patients and normal controls. Using 200 μL aliquots of the serum samples, we detected nine polyamines and eight steroids for which the concentrations varied in the ranges of 0.14–632.22 ng/mL with polyamines and 0.12–62.74 ng/mL with steroids. Large variations in both polyamine and steroid levels were observed between patients with breast cancer after treatment and normal controls. As shown in Table 3, all polyamines were higher in the patient groups, whereas most of the steroids were higher in the patient groups, except 11β-OHP. Among the metabolites we analyzed, it was confirmed that polyamines, androgen, and progesterone levels were similar to those of reference ranges [19,20,21,22].

### 2.5. Receiver Operating Characteristic Curve

**Figure 4:**
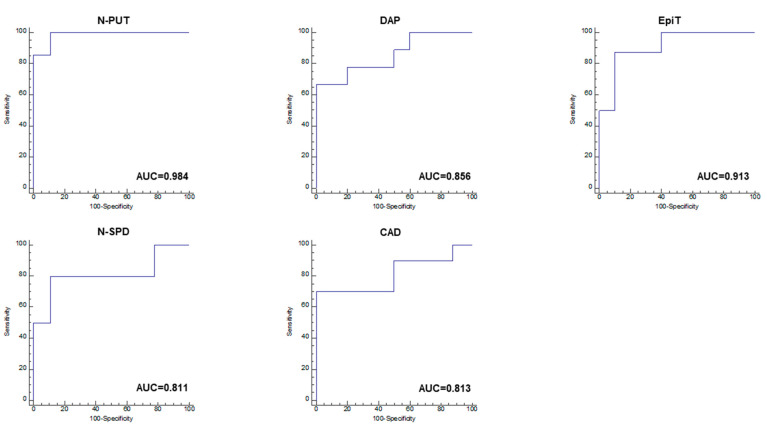
Univariate receiver operator characteristic (ROC) curve analyses for predicting biomarker performance in serum samples from treated breast cancer patients. Typical ROC curve plots of potential biomarkers with high-performance prediction are shown; several metabolites with AUC > 0.8 were observed. The ROC value of each metabolite was as follows: N-PUT, 0.984; N-SPD, 0.811; DAP, 0.856; CAD, 0.813; EpiT, 0.913 (N-PUT: *N*-acetyl putrescine; N-SPD: *N*-acetyl spermidine; DAP: 1,3-diaminopropane; CAD: cadaverine; EpiT: epitestosterone).

Receiver operating characteristic (ROC) curve analysis is generally performed to derive potential biomarkers. The results of ROC curve analysis were based on the results of univariate and multivariate statistical analyses that ensured the reliability of potential biomarkers for independent validation. Through t-test analysis, N-PUT, N-SPD, DAP, CAD, and EpiT were found to be significant markers, and the ROC curves for these five compounds were plotted (Figure 4). The area under the curve (AUC) for the remaining polyamines, namely N-SPD, DAP, CAD, and EpiT, was higher than 0.8, whereas the AUC for N-PUT and EpiT was higher than 0.9; therefore, it was possible to predict the potential markers.

All polyamines were higher in treated cancer patients than in normal controls. Since polyamines are involved in cell proliferation, polyamine levels increase in cancer patients. The results corroborate several studies conducted in human urine [23], serum [7], and saliva [24]. In particular, N-PUT, N-SPD, and DAP were significantly higher in patients, which correlated with other breast cancer research [7]. DAP has been reported to be associated with cell proliferation [25]. In addition, N-SPD and N-SPM were not detected in tissues of normal humans, whereas N-SPD was reported to increase rapidly in tissues of breast cancer patients [6]. In particular, acetylpolyamine is an important factor in breast cancer research, and our results are consistent with studies that revealed a correlation between intracellular polyamine levels and alterations in histone acetylation and deacetylation in normal and cancer cells [26,27]. Most of the steroids, except 11β-OHP, were increased in breast cancer patients. In particular, EpiT was significantly higher in the patient group. Although there are few studies on the correlation between breast cancer and EpiT, certain studies have reported that epitestosterone levels are five times higher in breast cysts [28].

## 3. Materials and Methods

### 3.1. Chemicals

Reference standards for nine polyamines and steroids were obtained from Sigma-Aldrich (St. Louis, MO, USA), Steraloids (Newport, RI, USA), and Tokyo Chemical Industry (Tokyo, Japan). The IS, d_3-_epitestosterone, and 1,6-diaminohexane were obtained from Sigma-Aldrich for calibration. For both calibration and QC, a commercially available blank serum sample (UTAK laboratories Inc, Valencia, CA, USA) was used. Sodium carbonate, sodium bicarbonate, formic acid (ACS reagent), and isobutyl chloroformate (derivatization solution) were obtained from Sigma-Aldrich. All high-performance liquid chromatography (HPLC)-grade organic solvents, including diethyl ether, acetonitrile, and methanol, were acquired from Burdick and Jackson (Muskegon, MI, USA). Deionized water (DW) was obtained using a Milli-Q purification system (Millipore, Billerica, MA, USA).

### 3.2. Preparation of Standard Solution

To prepare the reference standard stock solutions, polyamines and steroids were dissolved in methanol at a concentration of 1000 μg/mL. Working solutions were prepared using serial dilution with methanol at concentrations ranging from 100 μg/mL to 1 ng/mL. Moreover, 1,6-diaminohexane (DAH), one of the ISs, was dissolved in methanol at a concentration of 100 μg/mL. Working solutions were prepared using serial dilution with methanol at a concentration of 1 μg/mL. Further, EpiT-d3, another IS, was dissolved in acetonitrile at a concentration of 10 μg/mL. Working solutions were prepared using dilution with acetonitrile at a concentration of 1 μg/mL. All standard solutions were stored at −20 °C until further use.

### 3.3. Sample Information and Ethics Statement

Quantitative profiling of polyamines and steroids was conducted with 20 human serum samples from treated breast cancer patients (*n* = 10; aged 27 to 60 years, mean 51.5) and normal controls (*n* = 10; aged 27 to 59 years, mean 45.7). Serum samples were collected at the Yonsei University College of Medicine, and informed consent was obtained from each participant before collection. The study was approved by the institutional review board of Yonsei University College of Medicine (IRB No. 3-2017-0097). Participants were diagnosed with breast cancer of stage I–III and completed cancer treatment. Moreover, breast cancer patients completed cancer treatment, including surgery, adjuvant chemotherapy, and radiotherapy.

### 3.4. Sample Preparation

To remove proteins, the serum samples (200 μL) were added to 1 mL of DW at 60 °C for 20 min. After heating, 50 μL of the IS (1 µg/mL of DAH and EpiT-d_3_) was added. Thereafter, the pH values of the samples were adjusted to 9.0 with 1.0 M sodium carbonate buffer (25 µL), and the derivatization reagent (20 µL) was added. Then, the mixture was incubated at 35 °C for 15 min. After cooling, the solution was extracted twice with 2 mL of diethyl ether for 15 min along with shaking and centrifuged for 5 min at 1300× *g* using a Heraeus SEPATECH Varifuge 3.0, and the organic solvent was transferred to a new test tube. The entire organic layer was evaporated. The method followed in this study was the same as that described by Byun et al. [18]. The residue was reconstituted with 100 µL of MeOH, and a 5 µL aliquot was injected into the liquid chromatography–tandem mass spectrometry system.

### 3.5. Liquid Chromatography–Tandem Mass Spectrometry

LC-MS/MS analysis was performed using a Shiseido nanospace SI-2 HPLC system (OSAKA SODA, Osaka, Japan) coupled with a Thermo LTQ XL ion trap MS capable of electrospray ionization (Thermo, San Jose, CA, USA). Chromatographic separation was achieved using a Thermo Hypersil GOLD C18 column (150 × 2.1 mm, particle size: 3 µm) at a flow rate of 100 µL/min. A gradient eluent (A: 0.1% formic acid in 5% acetonitrile; B: 0.1% formic acid in 95% acetonitrile) was used. The gradient elution system was controlled as follows: 0 min, 50% B; 0–12 min, 50–95% B (hold for 5 min); 17–18 min, 95%–50% B. The gradient was then returned to the initial condition (50% B) and held for 10 min before running the next sample. The column and autosampler temperatures were maintained at 35 °C and 4 °C, respectively. The use of tandem MS systems is robust and sensitive. First, we acquired the full spectrum of the selected precursor. Moreover, we acquired the MS spectrum using a full scan in selected reaction monitoring (SRM) mode, and we chose the product ion value with a higher ion intensity parameter. For each analyte, the most abundant ion product was selected as the quantitation ion in SRM mode analysis. MS was performed under the following conditions: spray voltage = 5.0 kV; capillary temperature = 350 °C; sheath gas flow rate = 20 arb, auxiliary gas flow rate = 5 arb; capillary voltage = 32 V; tube lens voltage = 85 V; multipole 00 offset = −4.5V. All analytes were detected in positive ion SRM mode. The analytical conditions were optimized for each analyte (Table 4).

### 3.6. Validation

We carried out validation according the ICH guideline on bioanalytical method validation [29]. For validating the method, QC samples were prepared in commercial blank serum by spiking all target analytes at four different concentrations within their respective calibration ranges. Calibration standards and QC samples were prepared by adding a dilution of the stock analyte solution to the blank serum samples on every validation day. Further, the ion peak area of each metabolite was normalized by dividing it by the IS (e.g., polyamine groups-DAH; steroid groups-EpiT-d_3_).

Calibration curves were plotted with concentrations ranging from 1 to 5000 ng/mL for most polyamines (except N-SPM; range = 0.1 to 5000 ng/mL); further, T, EpiT, DHT, and PREG were plotted from 1 to 2000 ng/mL and 17α-OHP, 11β-OHP, A, and P4 were plotted from 0.1 to 2000 ng/mL. The linearity was evaluated using the correlation coefficient (R^2^) of the calibration curves. LOQ was defined as the lowest concentration with a signal-to-noise ratio greater than 10. Recovery was assessed to determine whether analyte compounds were lost during sample pretreatment. Overall recoveries were calculated by comparing the peak area ratios of analytes with the IS from all pretreatment steps versus those of only their derivatization steps. The matrix effect of urine samples due to endogenous substances compared to a standard solution was calculated as follows: (the ratio of spiked analyte standards and ISs in urine—the ratio of analytes and internal standards in blank urine)/(the ratio of the corresponding standard and IS in the standard solution) × 100 (%) [30]. Accuracy and precision were determined using QC samples at four concentrations, 10, 50, 500, and 1000 ng/mL, for most analytes, whereas analytes with an LOQ of 0.1 were measured at four concentrations of 1, 50, 500, and 1000 ng/mL. Accuracy was evaluated as the percent relative error (% bias), and precision was evaluated as the coefficient of variation (% CV). Intra-day validation was confirmed by analyzing three replicate samples, whereas inter-day validation was confirmed by analyzing the sample on three different days.

### 3.7. Statistical Testing and Data Processing

Polyamine and steroid concentrations were obtained from the calibration curves. Polyamines and steroid levels are expressed as the mean ± standard deviation (SD). We used Tune plus and Xcalibur for data analysis. Comparisons between normal controls and patients with breast cancer were performed using an independent samples Student’s t-test. For each of the measurements, the difference between groups was analyzed using a t-test followed by Bonferroni correction. The threshold of significance was set at *p* < 0.05. ROC curves to find the possible candidate biomarkers were plotted using MedCalc software (MedCalc Software, Mariakerke, Belgium). The threshold of significant markers was set to an AUC value of > 0.8.

## 4. Conclusions

In this study, a simultaneous analysis method using LC-MS/MS with isobutyl chloroformate derivatization was validated for serum polyamines and steroids. Polyamines and steroids have different functional groups; therefore, we derivatized polyamines with isobutyl chloroformate but not steroids. Combined quantitative profiling was performed, and sample preparation involved derivatization and an LLE step. This method was successful and reliable with satisfactory peak separation. Thus, we applied quantitative profiling of serum polyamines and steroids in age-matched breast cancer patients and normal controls and observed certain significant differences. Since we conducted the experiment with 10 pairs, it is not accurate, but through our simultaneous analysis method, we might be able to confirm the treatment effect for breast cancer patients. In our results, the clinical impact of several significant compounds showing clinical relevance is expected to be confirmed by extending the number of patients in the future. Although there are several studies on polyamines and steroids separately, our study is the first to analyze polyamines and steroids simultaneously. This method is advantageous as it makes use of a small quantity of samples and reduces the analysis time. In addition, this MS-based quantitative profiling method will be applicable to not only polyamine-related diseases, such as cancer, but also androgen-dependent diseases, such as male pattern baldness and benign prostatic hyperplasia.

## Figures and Tables

**Table 1:** Calibration range, linear regression equation, limit of quantification (LOQ), matrix effect, and recovery of polyamines and steroids.

Analytes	Calibration Range	Linear Regression Equation	Standard Errors of the Slope	Standard Errors of the Intercept	R^2^	LOQ	Matrix Effect (%)	Recovery (%)
N-PUT	1–5000	y = 0.0008x + 0.0986	1.42^−5^	0.03	0.998	1	101.4	107.4
CAD	1–5000	y = 0.0001x + 0.0718	6.07^−6^	0.01	0.995	1	83.1	87.9
N-SPM	0.1–5000	y = 0.084x − 0.6771	1.76^−4^	0.34	0.998	0.1	86.8	101.8
PUT	1–5000	y = 0.001x + 0.0949	7.90^−5^	0.15	0.992	1	115.5	98.2
SPD	1–5000	y = 0.01x − 0.6062	1.59^−3^	0.31	0.999	1	126.3	90.1
DAP	1–5000	y = 0.0013x + 0.0449	5.89^−5^	0.11	0.996	1	89.7	101.7
N-SPD	1–5000	y = 0.0067x + 0.419	3.76^−4^	0.73	0.990	1	118.4	123.6
N-CAD	1–5000	y = 0.0003x + 0.13	1.26^−5^	0.02	0.992	1	87.5	100.7
SPM	1–5000	y = 6E-05x + 0.0088	1.79^−5^	0.03	0.995	1	86.4	107.8
T	1–2000	y = 0.0032x + 0.1867	7.92^−5^	0.15	0.996	1	106.7	89.9
EpiT	1–2000	y = 0.0045x + 0.4609	1.59^−4^	0.31	0.993	1	103.4	100.4
DHT	1–2000	y = 0.0019x + 0.0017	6.13^−5^	0.12	0.994	1	82.1	104.7
PREG	1–2000	y = 0.0003x − 0.0244	1.11^−5^	0.02	0.991	1	79.3	97.7
17α-OHP	0.1–2000	y = 0.0115x + 0.1561	3.94^−4^	0.77	0.993	0.1	84.6	87.8
11β-OHP	0.1–2000	y = 0.0049x + 0.1846	1.83^−4^	0.36	0.992	0.1	88.6	91.8
A	0.1–2000	y = 0.0017x + 0.038	3.38^−5^	0.07	0.998	0.1	78.7	101.0
P4	0.1–2000	y = 0.0062x − 0.2893	3.01^−4^	0.58	0.994	0.1	100.9	96.7

N-PUT: *N*-acetyl putrescine; CAD: cadaverine; N-SPM: *N*-acetyl spermine; PUT: putrescine; SPD: spermidine; DAP: 1,3-diaminopropane; N-SPD: *N*-acetyl spermidine; N-CAD: *N*-acetyl cadaverine; SPM: spermine; T: testosterone; EpiT: epitestosterone; DHT: dihydrotestosterone; PREG: pregnenolone; 17α-OHP: 17α-hydroxyprogesterone; 11β-OHP: 11β-hydroxyprogesterone; A: androstenedione; P4: progesterone.

**Table 2:** Intra-day and inter-day validation of polyamines and steroids

Analytes	Spiked Concentration (ng/mL)	Intra-Day (*n* = 3)		Inter-Day (*n* = 3)	
		Accuracy	Precision	Accuracy	Precision
		(%Bias)	(%CV)	(%Bias)	(%CV)
N-PUT	10	91.2	8.1	88.1	11.3
	50	96.8	5.4	96.4	13.6
	500	110.4	7.0	92.9	18.5
	1000	104.1	7.0	106.7	2.6
CAD	10	86.5	13.0	93.8	13.4
	50	107.4	5.9	100.0	7.9
	500	95.4	11.1	103.2	5.2
	1000	92.1	9.2	102.5	9.8
N-SPM	1	104.0	16.9	104.6	19.2
	50	88.2	15.3	101.3	0.3
	500	113.6	6.2	110.5	3.1
	1000	112.8	4.8	118.3	2.9
PUT	10	99.0	10.5	107.3	18.3
	50	103.5	8.6	105.5	5.8
	500	99.1	15.3	115.1	3.2
	1000	103.4	15.4	102.0	17.0
SPD	10	105.8	16.3	96.2	19.7
	50	116.2	21.2	109.6	17.5
	500	93.8	18.3	92.0	6.8
	1000	98.6	19.6	104.7	11.5
DAP	10	108.6	17.1	94.3	16.4
	50	105.4	8.7	94.7	20.2
	500	101.2	1.7	106.6	10.1
	1000	103.9	6.2	93.6	6.6
N-SPD	10	108.5	1.6	106.5	18.3
	50	110.6	11.5	104.8	11.5
	500	104.3	6.5	105.6	13.6
	1000	114.5	2.7	106.3	16.5
N-CAD	10	95.5	13.9	115.6	4.1
	50	109.6	15.1	111.0	14.3
	500	110.5	9.6	90.7	12.4
	1000	97.5	13.5	90.3	9.5
SPM	10	91.5	14.9	92.4	0.7
	50	104.2	13.3	119.3	7.8
	500	104.3	10.6	87.8	6.9
	1000	103.5	12.2	98.4	16.4
T	10	104.9	12.5	106.8	17.6
	50	94.8	14.0	106.1	11.2
	500	104.0	2.4	94.6	2.5
	1000	100.7	9.0	100.5	8.1
EpiT	10	100.9	7.0	101.7	5.9
	50	106.8	9.0	102.1	6.4
	500	108.6	10.0	104.0	4.3
	1000	105.6	6.9	99.9	12.3
DHT	10	106.6	8.7	97.7	9.5
	50	114.3	6.5	90.2	11.9
	500	111.5	3.9	97.6	16.0
	1000	100.6	6.8	95.5	18.5
PREG	10	111.1	17.8	82.0	17.7
	50	92.4	0.6	96.6	7.5
	500	98.9	16.0	96.1	11.4
	1000	103.7	7.4	101.7	8.6
17α-OHP	1	101.5	17.1	107.9	8.4
	50	102.2	15.4	106.8	7.9
	500	109.1	13.2	100.4	3.8
	1000	96.4	1.2	95.0	1.2
11β-OHP	1	98.9	3.5	99.4	13.5
	50	95.0	21.8	108.0	4.7
	500	109.3	6.0	92.0	7.7
	1000	109.9	7.1	106.1	7.3
A	1	106.1	5.7	107.0	10.0
	50	96.9	6.9	94.8	10.2
	500	101.0	1.0	107.8	4.2
	1000	93.3	8.5	91.2	9.0
P4	1	99.9	19.7	101.0	1.7
	50	101.0	3.5	97.1	9.4
	500	91.0	13.1	98.3	8.6
	1000	110.8	5.3	103.0	18.1

N-PUT: *N*-acetyl putrescine; CAD: cadaverine; N-SPM: *N*-acetyl spermine; PUT: putrescine; SPD: spermidine; DAP: 1,3-diaminopropane; N-SPD: *N*-acetyl spermidine; N-CAD: *N*-acetyl cadaverine; SPM: spermine; T: testosterone; EpiT: epitestosterone; DHT: dihydrotestosterone; PREG: pregnenolone; 17α-OHP: 17α-hydroxyprogesterone; 11β-OHP: 11β-hydroxyprogesterone; A: androstenedione; P4: progesterone.

**Table 3:** Concentrations of nine polyamines and eight steroids in human serum samples of patients with breast cancer after treatment and normal controls (ng/mL).

	Normal Controls (*n* = 10)		Patients (*n* = 10)		*p* Value
	Mean ± SD	Median, Range	Mean ± SD	Median, Range	
*Polyamines*					
N-PUT	11.12 ± 4.34	11.09, 1.6–16.53	61.64 ± 58.88	46.57, 15.88–183.08	0.021
CAD	60.3 ± 27.33	66.35, 12.17–96.11	208.21 ± 169.71	174.62, 31.9–545.38	0.027
N-SPM	0.53 ± 0.45	0.45, 0.14–1.81	1.1 ± 1.13	0.82, 0.16–3.6	0.12
PUT	54.88 ± 70.74	16.3, 2.65–232.98	120.94 ± 170.91	38.52, 2.38–586.46	0.146
SPD	15.15 ± 20.73	4.88, 1.3–76.45	19.92 ± 22.53	13.62, 1.06–77.13	0.551
DAP	14.38 ± 6.08	14.99, 2.5–22.12	39.48 ± 21.64	36.79, 12.02–67.47	0.003
N-SPD	31.06 ± 15.91	31.05, 4.42–58.37	57.75 ± 25.66	58.35, 15.51–95.85	0.016
N-CAD	20.04 ± 16.63	16.45, 2.45–50.16	76.8 ± 84.72	39.11, 15.28–236.6	0.109
SPM	63 ± 48.54	60, 13.91–172.96	179.37 ± 207.84	76.83, 7.58–632.22	0.121
*Steroids*					
T	4.86 ± 3.44	3.52, 1.3–10.09	8.06 ± 6.24	6.52, 1.78–18.88	0.095
EpiT	12.64 ± 5.36	10.79, 7.51–24.55	28.74 ± 13.93	24.85, 12.39–53.79	0.004
DHT	2.35 ± 1.22	1.7, 1.6–3.76	2.71 ± 1.94	2.71, 1.34–4.08	0.809
PREG	32.98 ± 11.37	31.97, 16.16–47.4	44.87 ± 15.27	44.47, 23.56–62.74	0.106
17α-OHP	1.15 ± 1.21	0.73, 0.14–3.76	1.15 ± 1.35	0.71, 0.12–4.08	0.999
11β-OHP	2.12 ± 1.7	1.9, 0.62–5.08	1.78 ± 1.47	1.42, 0.42–4.6	0.698
A	1.41 ± 1.07	1.01, 0.29–3.29	2.73 ± 2.03	2.02, 0.68–6.73	0.09
P4	1.3 ± 0.87	1.05, 0.18–3.64	1.47 ± 0.82	1.34, 0.27–3.06	0.569

N-PUT: *N*-acetyl putrescine; CAD: cadaverine; N-SPM: *N*-acetyl spermine; PUT: putrescine; SPD: spermidine; DAP: 1,3-diaminopropane; N-SPD: *N*-acetyl spermidine; N-CAD: *N*-acetyl cadaverine; SPM: spermine; T: testosterone; EpiT: epitestosterone; DHT: dihydrotestosterone; PREG: pregnenolone; 17α-OHP: 17α-hydroxyprogesterone; 11β-OHP: 11β-hydroxyprogesterone; A: androstenedione; P4: progesterone.

**Table 4:** Optimized MS information for polyamines and steroids.

Compound	Abbreviation	Precursor Ion (*m/z*)	Product Ion (*m/z*)	Normalized Collision Energy (%)	Retention Time (min)
1,3-diaminopropane	DAP	275.0	201.1	22	9.3
Putrescine	PUT	289.0	215.0	28	10
Spermidine	SPD	446.1	372.3	35	14.6
Spermine	SPM	603.0	529.2	48	17.9
1,6-diaminohexane	DAH	317.0	243.0	27	12.45
Cadaverine	CAD	303.0	229.0	45	11.2
*N*-acetyl putrescine	N-PUT	231.0	157.0	28	4.8
*N*-acetyl spermidine	N-SPD	388.0	314.2	24	7.6
*N*-acetyl spermine	N-SPM	545.4	471.3	37	12.5
*N*-acetyl cadaverine	N-CAD	245.0	171.1	51	5.1
Testosterone	T	289.2	271.3	56	8.7
Dihydrotestosterone	DHT	291.2	255.3	24	11.1
Epitestosterone	EpiT	289.2	271.3	26	9.8
Epitestosterone-d_3_	EpiT-d3	292.2	256.4	35	9.7
Androstenedione	A	287.2	269.3	48	9.7
Pregnenolone	PREG	317.2	299.3	29	14.1
Progesterone	P4	315.2	297.3	30	13.9
17α-Hydroxyprogesterone	17α-OHP	331.2	313.3	22	9.8
11β-Hydroxyprogesterone	11β-OHP	331.2	313.3	29	9.2

## Data Availability

Not applicable.

## Sample Availability

Not available.
